# Responses in cognitive hierarchy games are correlated with academic performance and the cognitive reflection test

**DOI:** 10.3389/fpsyg.2023.1214534

**Published:** 2023-08-09

**Authors:** César Mantilla, Silvia Ortiz-Merchán

**Affiliations:** Economics Department, Universidad del Rosario, Bogotá, Colombia

**Keywords:** bounded rationality, lowest unique positive integer, beauty contest, university students, online proctored exams

## Abstract

Economics and Finance undergraduate students from four cohorts played LUPI, a game rewarding the person submitting the lowest unique positive integer, for a small bonus in an exam. Some months later, they played this game again with financial incentives and took a cognitive reflection test (CRT). We find that submitted responses to different configurations of LUPI are correlated with short-term (i.e., exam grade) and medium-term (i.e., final grade and GPA) academic performance, as well as the score in the CRT.

## 1. Introduction

Models of imperfect strategic thinking are essential tools in understanding bounded rationality. They explain out-of-equilibrium behavior and beliefs' formation in static settings (McKelvey and Palfrey, 1995; Nagel, 1995; Camerer et al., 2004; Goeree and Holt, 2004). These models apply to auctions, elections, and movie openings (McKelvey and Patty, 2006; Crawford and Iriberri, 2007; Brown et al., 2013; Crawford et al., 2013). Famous (and simple to explain) games employed to validate these models include the beauty contest, the 11–20 game, and the lowest unique positive integer–LUPI–game (Nagel, 1995; Östling et al., 2011; Arad and Rubinstein, 2012). Most of the evidence for these games comes from lab experiments and large-scale implementations in newspapers, magazines, and lottery companies (Bosch-Domenech et al., 2002; Östling et al., 2011). The former are insightful to learning from repeated decisions and multiple parameterizations in a controlled environment. The latter offer tests of bounded rationality with a more numerous and diverse sample.

Our study steps in between, linking responses in LUPI games conducted with students (at three different points in time) with their academic performance and results in a cognitive reflection test (CRT). The latter indicates the propensity to engage in analytical reasoning (Frederick, 2005; Pennycook and Rand, 2019) and is mildly correlated with creative thinking (Corgnet et al., 2016) and with academic achievement in secondary school (Gómez-Veiga et al., 2018). Four cohorts of students played a LUPI game that was added as a bonus question in an exam, and months later, they were invited to play two LUPI and beauty contest games with monetary incentives. We show a correlation between submitting responses more representative of equilibrium predictions and better academic performance in the short-term (exam grade) and the medium-term (course grade and GPA). Similarly, aligned with findings for the beauty contest (Gill and Prowse, 2016), decisions in the LUPI game that are more responsive to the strategic environment (i.e., a reduction in the number of contestants) are positively correlated with scores in the CRT, and with students' performance. Our results constitute an exhibit of external validity for games studying strategic and boundedly rational behavior.

## 2. Experimental paradigm

### 2.1. Implementation in the exams

We started to use the LUPI game as part of the preamble code in a practical econometrics exam that we aimed to “personalize” by randomly eliminating 20% of the sample in each student's dataset. To do so, we asked them for the last digit of their ID and to submit an additional integer by playing the LUPI game. The bonus question in the exam said:

*The choice of the randomizer inside the set.seed() command is a bonus. Your answer should be in the variable* number_bonus*. You must choose an integer number between 1 and 100, knowing that whoever chooses the least non-repeated positive integer in each class will have a bonus of +0.3 on the exam*.

*You face a trade-off when choosing this number: the higher the number, the lower the chance that it will be repeated (more chance of winning). However, the higher the number, the higher the chance that someone else will say a lower number (less chance of winning)*.

LUPI decreases the incentives to collude because two students submitting the same number will, by definition, lose. By offering to the winner of LUPI in each class a bonus of +0.3 in their final grade, they may increase by 6% of their maximum attainable grade (9.25% of the average grade in the exams). By definition, only one student per class could receive this bonus. Since the average number of students in each class was 35.4, in expectation, this reward has minimal impact on the grades in the class despite being individually appealing.

Two hundred and forty-eight students from Universidad del Rosario in Bogotá (Colombia) took the Introductory Econometrics class in four different academic terms between 2020 and 2021. From them, 109 students played LUPI in two exams, and 133 played it in a single exam. The remaining students played LUPI at least three times because they took the class twice. In total, we have 300 observations in which we can link the grade in the exam with their play in the LUPI game, which we will call *LUPI-Exam* hereafter.

### 2.2. Implementation of the incentivized game

We conducted an incentivized asynchronous study one month before the last cohort finished their Econometrics course. We sent an e-mail to all the current and former students, inviting them to play LUPI and a similar game with economic incentives. We informed them that we were expecting to have 160 participants to play four games: (i) LUPI with all the other contestants, *LUPI-All* hereafter; (ii) LUPI in groups of 40 participants, *LUPI-40*; (iii) a Beauty Contest game with all the other contestants, *BC-All*; and (iv) a Beauty Contest game in groups of 40, *BC-40*. The winner in each game with all contestants earned COP 150,000, whereas the winner in each game in groups of 40 earned COP 40,000. These prizes were equivalent to 40 and 10.5 USD, respectively. In the two beauty contest games, participants could submit a number between 1 and 100; the winner was the person closest to two-thirds of the average. We also asked participants to complete a three-question cognitive reflection test and to report their average GPA. Supplementary material 4, 5 include the full protocol in English and Spanish, respectively.

We sent 248 survey invitations and gave participants thirteen days to complete it. We obtained 113 responses, the sample size for this second analysis. Supplementary material 1 describes the subsets of observations in the different analyses. The selection equation reported in Supplementary Table 1 reveals that former students were less likely to participate (−15 percentage points), and those obtaining a higher grade in the course were more likely to enter the incentivized study. Given this evidence of non-random selection, we conjecture that the coefficients reported in Section 3.2 constitute a lower bound due to the positive correlation between students' academic performance and strategic responsiveness in the LUPI games.

### 2.3. Hypotheses

**Figure 1:**
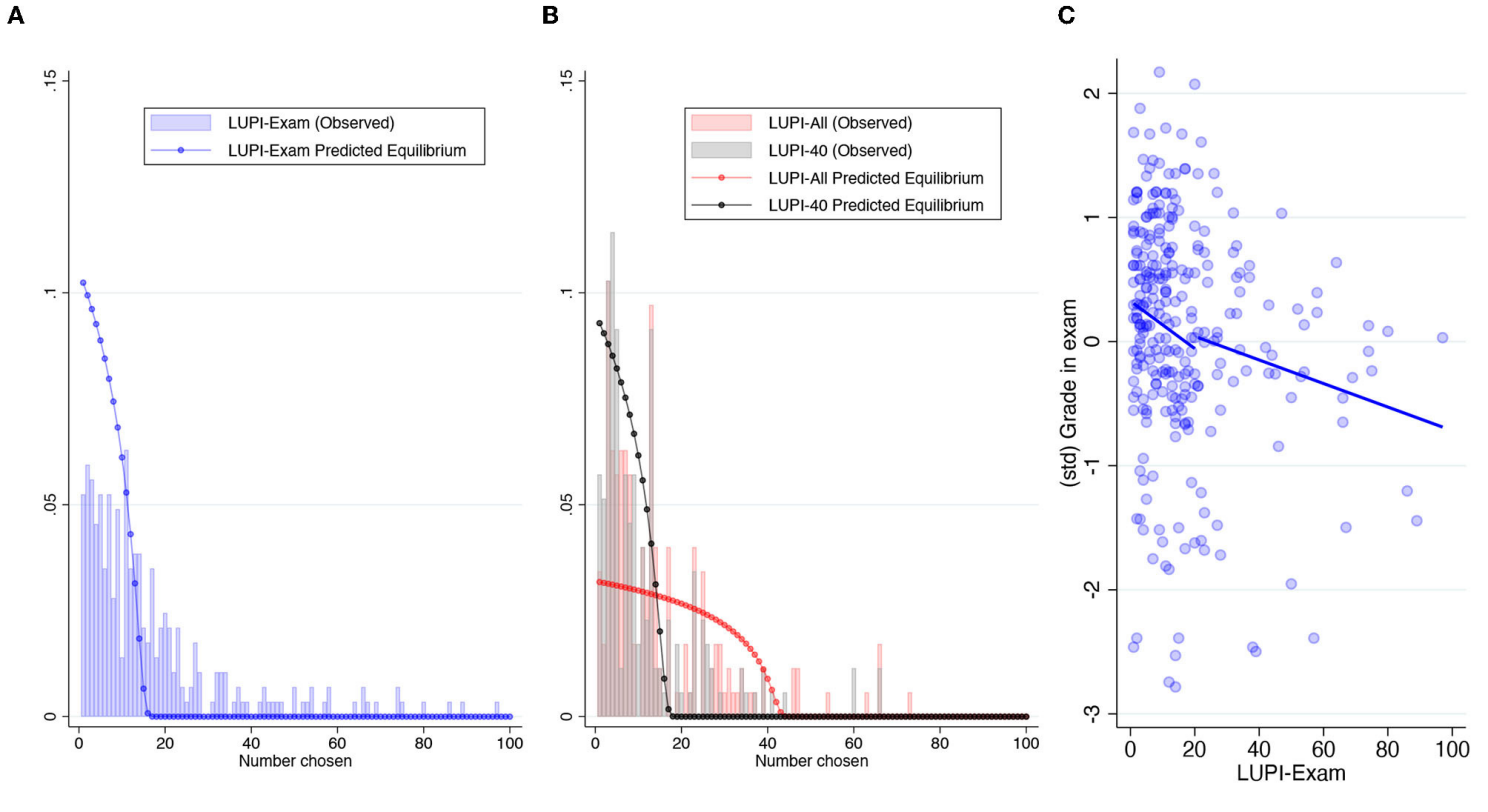
Distribution of submissions in the LUPI game. **(A)** LUPI distribution exams. **(B)** LUPI distribution incentivized games. **(C)** Correlation between LUPI-exam and exam grade.

Following Östling et al. (2011), LUPI games have a mixed strategy equilibrium in which the predicted frequencies of play monotonically as the submitted numbers increase. The distribution depends on the number of players (*n*) and the number of choices (*k*), which is 100 in all three games. In *LUPI-Exam* (*n* = 35), the probability of choosing 1 is 10.2%. This probability decreases fast enough, to the point that 15 is played with 0.6% of probability (and the cumulative probability is already 99.9%). The distribution of equilibrium play is very similar in the *LUPI-40* (*n* = 40). The probability of choosing 1 is 9.3%, and the first number played with < 1% of probability in equilibrium is 16 (with a cumulative probability of 99.8%). The *LUPI-All* (*n* = 160) has a different pattern because *n*>*k*. The probability of choosing 1 is 3.17%, and it decreases at a slower pace than in the other two games: the first number played with < 1% of probability in equilibrium is 40 (with a cumulative probability of 99.0%). Figures 1A, B allow us to compare these predictions with actual play, while Supplementary Figure 2 lets us compare the predictions for the three games.

Our first hypothesis relates to the responses in the *LUPI-Exam* and its correlation with the exam performance:

**H1: The submitted number in the** ***LUPI-Exam*** **is negatively correlated with the exam grade**.

In this game, most of the support of equilibrium play is accumulated in the first eleven digits (90%). Hence, we expect that students engaging in more cognitively demanding strategies (i.e., that make them close to an equilibrium play) also have better performance in the exam. There may be two different channels that we cannot distinguish, reinforcing that our result is merely a correlation. First, students engaging in more analytical thinking to solve the exam may also be more likely to submit a more strategic choice in the LUPI game. Second, it may also be that students expecting to perform better in the exam see it as less costly to devote some minutes of the exam to think about the *LUPI-Exam* more thoroughly.

Our second hypothesis focuses on the additional information from the incentivized stage of our study. It relates to how, combining the responses from multiple LUPI games, they can be informative of a correlation between CRT score and medium-term measures of academic performance:

**H2: The difference** ***LUPI-All***- ***LUPI-40*** **is positively correlated with the CRT score, the grade obtained in the econometrics course, and the GPA**.

To see why we focus on the difference in the responses between these two games, recall that the choice set is the same (*k* = 100) in the *LUPI-All* and *LUPI-40* games, but the number of contestants differs. Consequently, participants more engaged in strategic behavior would react to this change in the decision environment by shifting their choice to the left. This can be seen more clearly in the right panel in Supplementary Figure 2, showing the differences in cumulative probabilities of equilibrium play between *LUPI-All* and *LUPI-40*. We extend our intuition from H1 regarding the connection between responses in LUPI games and academic performance to medium-term outcomes, such as the final grade in the Econometrics course and the GPA, as representative of academic performance overall. For the CRT as a relevant outcome, the connection is more direct: the test measures the propensity to engage in analytical reasoning, which is also required to submit a successful response (i.e., belonging to the more likely choices in equilibrium) in the LUPI games. After this explanation, an extension of H1 is that the negative correlations would also hold for our additional outcome variables and the response to *LUPI-All*.

Our third hypothesis focuses on the relationship between the two LUPI games with similar equilibrium play, but differing in the timing of the question and the nature of incentives:

**H3: The difference** ***LUPI-Exam***- ***LUPI-40*** **is positively correlated with the CRT score, the grade obtained in the econometrics course, and the GPA**.

Whereas H2 explores how the responses to changes in the strategic environment correlate with the outcomes of interest, in H3 we explore whether there are some learning effects in playing these games that may correlate with these outcomes. Recall from Supplementary Figure 2 that the probability distribution of equilibrium play is very similar in the *LUPI-Exam* and *LUPI-40* games. Any shift in the distribution between the first and the second LUPI game in small groups (i.e., a positive difference) would suggest some learning.

## 3. Results

### 3.1. LUPI game in the exams

Figure 1A displays the resulting skewed distribution. The median response was 12, and the mean was 20.7. The larger mean also obeys to a peak in 101. This was the default choice, suggesting that 4.3% of students skipped the LUPI game in their exam. The empirical distribution reveals larger probabilities of play for two-digit choices compared to the equilibrium prediction, suggesting a high frequency of suboptimal play (e.g., there should be no choices above the total number of participants). This result differs from Östling et al.'s 2011 outcomes with small *n* (27) and repeated interactions, where the frequencies of play resemble more the equilibrium prediction (cf. Figure 9 in their study). We thus argue that the one-shot nature of our setting maintained the probability of choosing two-digit numbers relatively high.

The high frequency of suboptimal responses provides more variance for studying the correlation between *LUPI-Exam* responses and exam performance. Table 1 displays the OLS results for a regression in which *LUPI-Exam* is the single predictor of the standardized exam's grade. The coefficient in column 1 implies that participants submitting a number between 1 and 5 (i.e., the bottom quartile) performed at least 0.1 standard deviations higher in the exam than a student submitting 22 or a larger number (i.e., the third quartile). This magnitude is non-negligible. It is about one-third of the effect in a final exam from an educational intervention referring students to tutoring for extra academic support (0.29 standard deviations, see Gordanier et al., 2019). Also, it is about one-third of the average effect on student's performance from interventions enhancing critical thinking (a standardized average effect of 0.33 in a meta-analysis including 341 standardized effect sizes, see Abrami et al., 2015), a relevant comparison since critical thinking can be fostered by the strategic analysis required in LUPI games.

**Table 1:** OLS regression for the standardized exam grade based on the submitted positive integer (PI).

	**Standardized exam grade**	
	**(1)**	**(2)**
LUPI-exam	−0.006^**^	−0.010^***^
	(0.002)	(0.003)
Default PI (over 100)		0.719^*^
		(0.384)
Constant	0.195^***^	0.254^***^
	(0.074)	(0.077)
Observations	300	300
*R*-squared	0.025	0.037

Standard errors clustered at the student level in parentheses. ^***^p < 0.01, ^**^p < 0.05, ^*^p < 0.1.

In column 2, we added a categorical variable indicating submissions above 100 (i.e., those who did not change the default). This specification increases the precision of the coefficient of interest. Figure 1C validates the reported negative correlation. Moreover, splitting the prediction line shows that the correlation is negative before and after the mean *LUPI-Exam* response. This pattern suggests that the correlation is not driven by a specific subgroup (e.g., only by those with the worst grades that did not fully understood the game). Summing up, these results provide evidence in favor of H1.

### 3.2. LUPI games with incentives

In the *LUPI-All*, the median and mean responses were 11 and 14.9, respectively. The winner was 19. In the *LUPI-40*, the median and mean responses decreased to 8 and 11.6 (with winning numbers 2, 3, and 9). On average, participants best-responded to the fewer contestants in *LUPI-40* by submitting a lower number: with only one-third of the contestants in *LUPI-All*, they correctly anticipated that the distribution of responses shifted to the left. The average and median differences between *LUPI-All* and *LUPI-40* were 3.25 and 2. Still, 16% of participants submitted the same number in both games, and 29% submitted a higher number in *LUPI-40*. Perhaps due to this 45% of participants who did not adjust their response toward a smaller number when there were fewer competitors, the comparison of the empirical distributions (exact *p*−value from a Kolmogorov-Smirnov test) between *LUPI-All* and *LUPI-40* is not statistically significant (*p* = 0.056). Besides, the other comparisons between distributions reveal no differences between *LUPI-Exam* and *LUPI-All* (*p* = 0.285) despite the latter having a larger number of participants in the game. This is evidence of some learning in playing LUPI games reinforced by the comparison between *LUPI-Exam* and *LUPI-40* (*p* < 0.001). Despite the blue and black predictions in Figure 1 being almost identical, the gray distribution is more leaned to the left.

*LUPI-All* and its difference with *LUPI-40* are covariates in the OLS regressions reported in Table 2, explaining three outcomes: CRT score, the standardized grade obtained at the end of the Econometrics course, and the self-reported GPA. We also add the gender of the student as a control variable, given the existing performance differences in the CRT (Ring et al., 2016; Brañas-Garza et al., 2019). The odd columns reveal that submitting higher numbers in *LUPI-All* is correlated with a lower CRT score, a lower final grade in the Econometrics course, and a lower GPA. These results expand the evidence of a correlation between LUPI play academic outcomes, validated in H1, to the CRT and medium-term academic outcomes.

**Table 2:** OLS regression for medium-term outcomes: CRT score, grade in econometrics, and self-reported GPA.

**Variables**	**Score in CRT**		**Course grade (std)**		**GPA**	
	**(1)**	**(2)**	**(3)**	**(4)**	**(5)**	**(6)**
LUPI-All	−40.024^**^	−0.029^**^	−0.013^*^	−0.016^**^	−0.005^*^	−0.007^**^
	(0.010)	(0.012)	(0.007)	(0.007)	(0.003)	(0.003)
LUPI-All - LUPI-40	0.030^**^	0.041^**^	0.003	0.011	0.005	0.007
	(0.015)	(0.017)	(0.010)	(0.011)	(0.004)	(0.004)
LUPI-Exam - LUPI-40		−0.008		−0.001		−0.001
		(0.006)		(0.004)		(0.001)
Constant	2.273^***^	2.470^***^	0.477^***^	0.513^***^	4.064^***^	4.127^***^
	(0.169)	(0.201)	(0.114)	(0.125)	(0.046)	(0.054)
Observations	113	92	99	92	108	87
R-squared	0.083	0.100	0.046	0.055	0.056	0.066
Mean VIF	1.48	1.37	1.45	1.37	1.49	1.37

Gender of the student (=1 if female) is a control omitted from the output. It is statistically significant only in model (1). The coefficient is −0.547 with a standard error 0.239. Mean variance inflation factors (VIFs) reported for each regression. The largest individual VIF was 1.73. Standard errors in parentheses. ^***^p < 0.01, ^**^p < 0.05, ^*^p < 0.1.

Regarding the difference between *LUPI-All* and *LUPI-40*, its positive coefficient suggests that best responding to changes in the strategic environment (i.e., submitting lower numbers with fewer contestants) is correlated with the propensity to engage in analytical thinking (i.e., the CRT score) but not with medium-term academic outcomes. We thus have partial support for H2. We validate this hypothesis only for the CRT, a closely related outcome whose measure has the same nature in terms of timing (two tests measuring immediate performance rather than longer-term outcomes).

Supplementary Table 2 reports the regression outcomes when the difference *LUPI-All*- *LUPI-40* is excluded from the regression. Likelihood-ratio tests reveal that adding this variable improves the fit for the CRT (*p* = 0.039), but not for the course grade (*p* = 0.73) nor the GPA (*p* = 0.24). Hence, the additional information from comparing responses between the two incentivized LUPI games seems to operate mainly for the CRT.

We left for last the difference between *LUPI-Exam* and *LUPI-40*, added in the even columns of Table 2. This difference has a median 3 and a mean 10.4, suggesting some learning for a similar strategic environment between the initial LUPI game and the incentivized *LUPI-40*. This variable is not statistically significant. Thus, we do not find support for H3. A plausible alternative explanation for this lack of significance is that the added covariate was highly correlated with the other two. We argue that this is not the case for two reasons. First, Supplementary material 3 reports a correlational analysis across LUPI games, revealing that *LUPI-Exam* had low correlations with the other LUPI games. Second, at the bottom of Table 2, we show that the variance inflation factors, helpful to detect collinearity problems, were low compared to the critical value of 10.

### 3.3. Does LUPI offer any advantage, in this context, with respect to the beauty contest?

Recall that, in the incentivized stage, participants also submitted their strategies for two Beauty Contest games (BCG). This subsection aims to compare the goodness of fit between models using LUPI responses, BC responses, and both.

Table 3 reports these results, including the AIC measure of goodness of fit at the bottom. We can take the lowest AIC for each outcome *i* and compute its difference with model *j*, which we call Δ_*j*−*i*_. According to the rule of thumb described in Burnham and Anderson (2004), if Δ_*j*−*i*_ ≤ 2, model *j* has as much support as model *i*. We can draw two conclusions. First, only Δ_2 − 1_ and Δ_3 − 1_ are >2. This is consistent with larger and more statistically significant coefficients for LUPI compared to the BC, but only for the CRT score. Hence, this is the only outcome in which responses in the LUPI games could be more informative than those for the BC games. This result is aligned with a game form recognition problem for the BCG (Chou et al., 2009), where hints regarding the “spatial” meaning of a fraction of the average help lowering responses. We conjecture that not having to compute averages aids this game form recognition, as occurs in LUPI games and the “battle” variation of the BC game proposed by Chou et al. (2009). Second, adding all games to the regression [*cf* ., models (3), (6), and (9)] does not improve the model's goodness of fit for any outcome. Hence, both games capture similar information from strategic responses, so they are sufficiently correlated to yield a null gain of information when putting all four games together.

**Table 3:** OLS predictions of CRT score and academic outcomes with LUPI and the beauty contest responses.

	**(1)**	**(2)**	**(3)**	**(4)**	**(5)**	**(6)**	**(7)**	**(8)**	**(9)**
**Variables**	**Score in CRT**			**Course grade (std)**			**GPA**		
LUPI-All	−0.024^**^		−0.019	−0.013^*^		−0.011	−0.005^*^		−0.004
	(0.010)		(0.011)	(0.007)		(0.008)	(0.003)		(0.003)
LUPI-All - LUPI-40	0.030^**^		0.030^*^	0.003		0.005	0.005		0.005
	(0.015)		(0.015)	(0.010)		(0.010)	(0.004)		(0.004)
BC-All		−0.014^*^	−0.011		−0.011^**^	−0.007		−0.003	−0.002
		(0.008)	(0.009)		(0.005)	(0.006)		(0.002)	(0.002)
BC-All - BC-40		0.002	0.008		0.010	0.011		0.003	0.004
		(0.013)	(0.013)		(0.009)	(0.009)		(0.003)	(0.003)
Constant	2.633^***^	2.647^***^	2.771^***^	0.518^***^	0.520^***^	0.616^***^	4.019^***^	4.013^***^	4.047^***^
	(0.229)	(0.235)	(0.253)	(0.160)	(0.164)	(0.177)	(0.062)	(0.063)	(0.068)
Observations	113	113	113	99	99	99	108	108	108
R-squared	0.083	0.062	0.097	0.046	0.049	0.070	0.056	0.052	0.073
AIC	364.19	366.76	366.42	233.77	233.45	235.28	62.66	63.13	64.66

Gender of the student (= 1 if female) is a control omitted from the output. Standard errors in parentheses. ^***^p < 0.01, ^**^p < 0.05, ^*^p < 0.1.

## 4. Conclusion

We implemented LUPI with four cohorts of economic undergraduate students and showed that their choices are correlated with academic outcomes. Beyond the external validity for cognitive hierarchy games, LUPI has two advantages for implementations outside the lab. First, the instructions are simpler to follow compared to the beauty contest: selecting the lowest non-repeated integer sounds simpler than computing an expected average of responses and applying fractions. Second, LUPI creates incentives to avoid choosing the same number from others. We exploited the latter feature in a remotely proctored exam to personalize the students' datasets, making fraud attempts more costly.

We also advocate for using multiple cognitive hierarchy games into the same instrument, as they help improve the exploration of analytical reasoning. While Hanaki et al. (2019) employs multiple beauty contests to infer the participant's rationality in changing strategic environments, we believe that multiple LUPI games may also be helpful to study analytical and critical thinking with games having more straightforward instructions, as suggested in Chou et al. (2009).

From a broader perspective, LUPI games can be used as examples to develop critical thinking in Economics students motivating their abstract thought. Siegfried and Colander (2022) recently provided a list of concepts where we, as Economics instructors, should help develop critical thinking. Whereas, the list is extensive, concepts such as *comparative advantage* and *unintended consequence* require to develop priors about what the others may gain or the other may do, which can be reinforced through simple abstract games. This list also includes the need to understand the differences between *correlation and causation*, an essential goal in an Econometrics course that connects us to the need to develop the students' critical thinking abilities. In a related paper, commenting Siegfried and Colander's (2022) piece, List (2022) acknowledges that critical thinking is costly and makes a parallel with the dual-process approach to cognition: the more reflective nature of “slow” thinking makes it costly, so we need to guide students in how to slow-down their thinking. Thought experiments, with individual choices followed by group discussions, may help.

## Data availability statement

The datasets presented in this study can be found in online repositories. The names of the repository/repositories and accession number(s) can be found below: https://osf.io/8jekv/.

## Ethics statement

The studies involving human participants were reviewed and approved by Sala de Ciencias Sociales-Comité de Ética en Investigación de la Universidad del Rosario. The patients/participants provided their written informed consent to participate in this study.

## Author contributions

CM and SO-M equally contributed to the conceptualization, methodology, and formal analysis. CM contributed with the writing, visualization, and funding acquisition. SO-M contributed with the software programming and data curation. All authors contributed to the article and approved the submitted version.
