# Supplementary material for: Responses in cognitive hierarchy games are correlated with academic performance and the cognitive reflection test
Source: Front Psychol. 2023 Aug 9;14:1214534. doi: 10.3389/fpsyg.2023.1214534 (PMC10445122; doi:10.3389/fpsyg.2023.1214534)
Supplement: Supplementary file 1 [file Presentation_1.PDF]

Supplementary online material

Responses in cognitive hierarchy games are correlated with academic performance and the cognitive reflection test

César Mantilla[1], and Silvia Ortíz-Merchán[2]

Replication material

The data and code to replicate all the tables and figures from the paper can be found at: `https://osf.io/8jekv/?view_only=b4d88553a08e4d63bc58e3a0b33debb2`

SM.1 Explanation for the observations per regression

Figure SM.1 seeks to explain the differences in the number of observations across the regressions reported in the main text and in this supplementary material. These differences are explained by the timing of our data collection exercise: some students only submitted a response to the *LUPI-Exam* (the purple areas), some did it only in the incentivized stage, providing responses for the *LUPI-All* and *LUPI-40* (the orange area), and some replied to both (the blue areas). Moreover, some students submitted a response to the *LUPI-Exam* more than once (light blue and light purple areas), so they need to be excluded from the regressions in which the nature of the analysis limits us to one observation per participant (i.e., all but Table 1).

The top legend explains which observations were employed in each table, whereas the bottom legend reports the number of observations in each colored area. Regarding the orange area, these 36 observations can be further divided: 25 correspond to participants who also replied to the *LUPI-Exam*, but did not authorize the use of this information, nor their GPA and final grade; the remaining 11 only participated in the incentivized second stage. Since participation was by invitation, they probably took the exam at a different time, so they were not invited to participate in the *LUPI-Exam* game. The gray area corresponds to students who did not participate in any of our exercises but are included for completeness. Since the *LUPI-Exam* game was conducted during the pandemic, these 36 empty observations correspond to students who dropped the class very early (i.e., before the exams) or that they took the exam at a different moment (e.g., a replacement exam for medical reasons). We still sent the invitations for the incentivized second stage to the whole class.

Finally, in Tables 2, 3, and SM.2, the number of observations also varies across outcomes. The reason is that not all students authorized us to use their information regarding their final grade or their GPA.

[1]Department of Economics, Universidad del Rosario.
[2]Department of Economics, Universidad del Rosario.

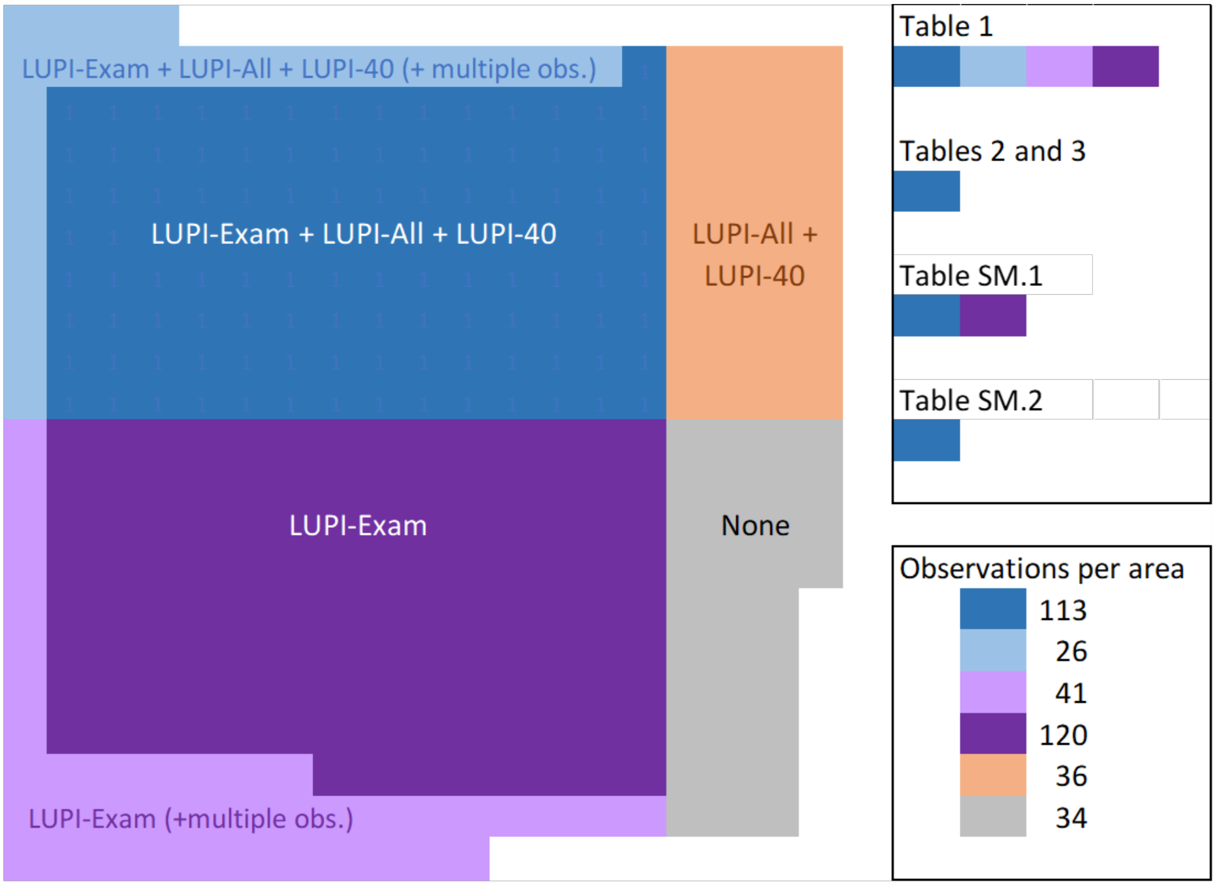

Figure SM.1: Observations types per colored area.

SM.2 Additional tables and figures

Table SM.1: Selection into the incentivized game (=1 if played the second part, =0 otherwise).

VARIABLES	(1) OLS	(2) Margins (logit)
Semesters since took class = 1	-0.157	-0.159*
	(0.0962)	(0.0953)
Semesters since took class = 2	-0.177**	-0.173**
	(0.0826)	(0.0811)
Semesters since took class = 3	-0.126	-0.126
	(0.0902)	(0.0915)
Final grade (standardized)	0.133***	0.140***
	(0.0299)	(0.0331)
Female student	0.0565	0.0565
	(0.0644)	(0.0635)
Constant	0.511***	
	(0.0695)	
Observations	233	233
R-squared	0.081	

Note: Standard errors in parentheses. *** $p < 0.01$, ** $p < 0.05$, * $p < 0.1$.

Table SM.2: OLS Predictions of CRT score and academic outcomes with *LUPI-ALL* as unique covariate.

VARIABLES	(1)<br>Score in CRT	(2)<br>Course grade (std)	(3)<br>GPA
LUPI-All	-0.012	-0.012**	-0.003
	(0.008)	(0.006)	(0.002)
Female student	-0.537**	-0.059	0.073
	(0.242)	(0.165)	(0.065)
Constant	2.548***	0.509***	4.005***
	(0.228)	(0.157)	(0.061)
Observations	113	99	108
R-squared	0.048	0.045	0.044

Note: Standard errors in parentheses. *** $p < 0.01$, ** $p < 0.05$, * $p < 0.1$.

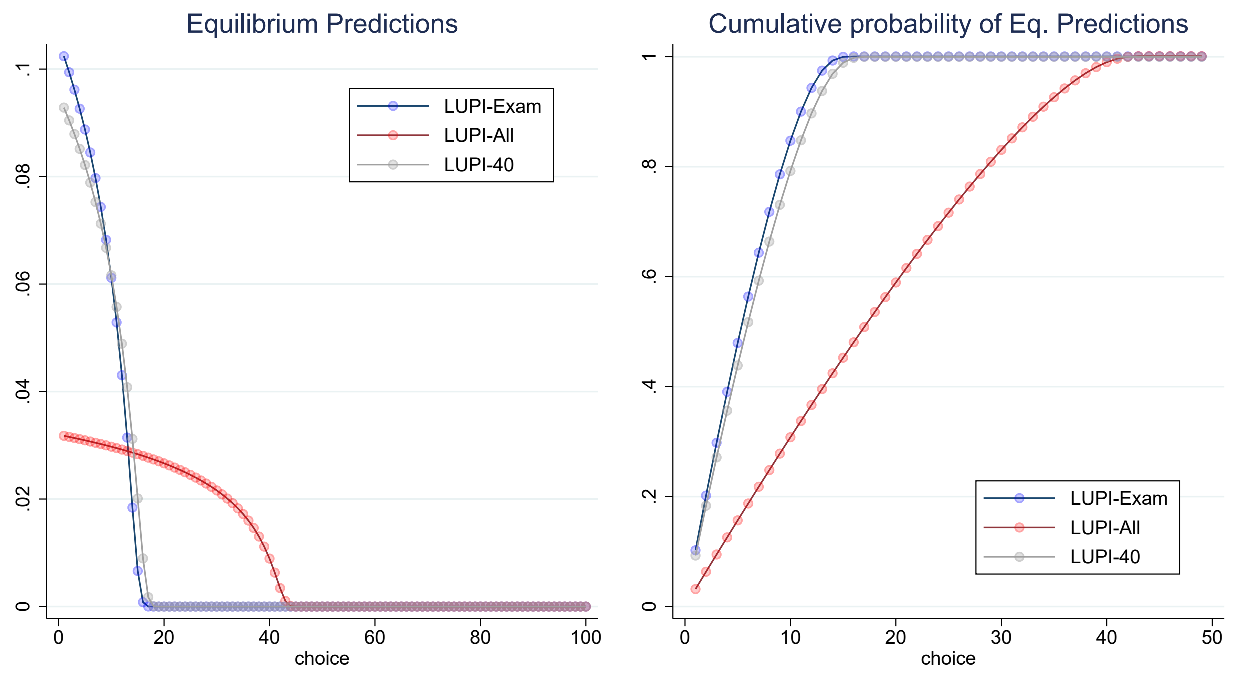

Figure SM.2: Equilibrium predictions for all the three LUPI games.

SM.3 Comparisons between responses to the cognitive hierarchy games and their explanatory power

Although the manuscript focuses on the LUPI game, in the incentivized second stage of our study we also collected data on the beauty contest. The purpose of this section is to provide more details on the comparisons between the two families of cognitive hierarchy games.

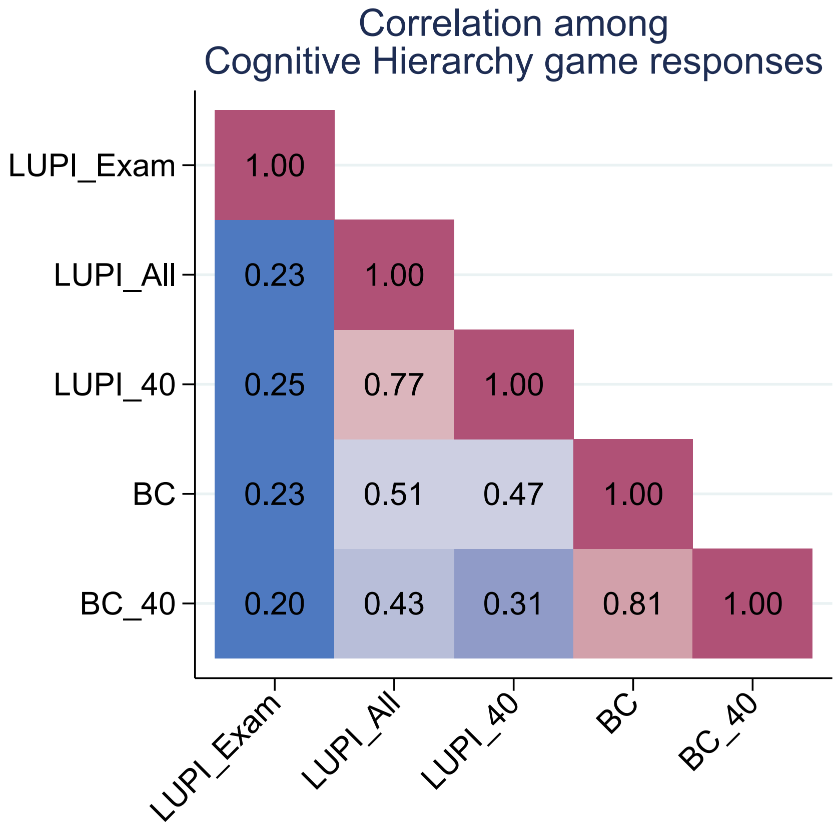

Figure SM.3: Matrix of pairwise Pearson's correlations between responses for each participant. $p-$values are below 0.001 for all correlations above 0.25. $p-$values for the blue cells in the first column are, in descending order, 0.030, 0.017, 0.027, and 0.061.

We start by showing the Pearson pairwise correlations in the responses to the five games, depicted in Figure SM.3. The different colored areas help us identify three results. First, the correlation of the *LUPI-Exam* game with the other four games is relatively low (between 0.20 and 0.25), though statistically significant (see the Figure SM.3's note for the report of $p-$values). Second, the most significant correlations appear for games of the same nature with different group sizes that were collected at the same time: *LUPI-All* with *LUPI-40* (0.77), and *BC-All* with *BC-40* (0.81).

Third, incentivized cross-game correlations (i.e., LUPI and BC measured in the same stage) are stronger than within-game correlations measured at two moments in time (i.e., the correlation for *LUPI-Exam* with *LUPI-All* and *LUPI-40*).

These correlations validate that models in Table 2 do not have multicollinearity problems, as was already suggested by the reported VIFs. That is, the correlation between *LUPI-All* and *LUPI-40* could have been too high and that would explain the lack of significance of their difference in models (3) to (6). In Table 3, on the other hand, the simultaneous inclusion of the responses to the LUPI and BC games reduces the individual statistical significance of coefficients despite the slight overall increase in joint significance.

SM.4 Translated protocol of the incentivized experiment

Strategic Thinking and Exams under Remote Learning

Do you think you can choose the smallest integer without repetition? Participate in this study on strategic games, academic performance, and attitudes towards virtual exams.

Informed Consent

You will participate again in the LUPI (Lowest Unique Positive Integer) game, an activity you completed in at least one of your Introductory Econometrics exams. You will also participate in another similar game.

The entire test will take about 15 minutes or less. You will play both games, in large groups and small groups, for a total of four of the games. By taking part in this test, you can participate for four different prizes, one for each version of the game. Games with all contestants pay $150,000 to the winner, and games in groups of 40 pay $40,000 to the winner of each pool.

Your participation is voluntary, and you can leave the test at any time, without any penalty or prejudice. In addition to the information collected in this test, we will request the use of your results in the Basic Econometrics exam where you played LUPI, specifically your response to the game, and your test scores.

Confidentiality: The information collected in this survey may be published in a scientific report or article. Your personal data will not be shared with third parties, and will be used exclusively for the purpose of relating your previous answers to the current ones and to be able to contact you in case you are one of the winners. Subsequently, this information will be completely anonymized, and may be stored by researchers to be used in future studies.

This test is part of a research project on attitudes towards virtual exams, academic performance, and other tests that require cognitive effort. The researcher in charge is César Mantilla from the Universidad del Rosario. If you have questions about this test, please send an email to cesar.mantilla@urosario.edu.co.

Draw of winners: Responses to the games will be received until October 28 at 7:00 pm. Subsequently, small groups will be randomly formed. With this information, the winners will be computed and published, together with the randomization code.

Please indicate below that you have read the information shown above, and that you wish to participate in this online test.

I have read the information shown above, and I wish to participate in this online test. I agree to the following treatment of my information:

☐ I approve the use of my information that is collected in this test and previously in class (in-game answer and exam grade).

☐ I approve of the use of my information that is collected in this test, but not my previous information in class (in-game answer and exam grade).

Game #1 – Choose the lowest unique positive integer in large groups

You must choose any integer you want between 1 and 100, knowing that whoever chooses the **smallest non-repeated positive number** will be the winner.

To choose the number you face a *trade-off*: the higher the number, the lower the chance that it will be repeated (more chances of winning), but also the higher the number, the greater chances that someone else will say a lower number (less chances of winning).

For this game, you will compete against the total number of people who enter this experiment. We have sent the invitation to **240** people and we hope that around **160** will respond to the activity.

The winner of Game #1 will win $150,000. Your answer:

Game #2 – Choose the lowest unique positive integer in small groups

The instructions are similar to the previous game.

You must choose any integer you want between 1 and 100, knowing that whoever chooses **the smallest non-repeated positive number** will be the winner.

To choose the number you face a *trade-off*: the higher the number, the lower the chance that it will be repeated (more chances of winning), but also the higher the number, the greater chances that someone else will say a lower number (less chances of winning).

For this game, you will be assigned to a group of **40 people**, and you will compete against them.

The winner of Game #2 in your group will win $40,000. Your answer:

Game #3 – Choose the average number of others in large groups

You must choose any integer you want between 1 and 100, knowing that whoever chooses the number **closest to 2/3 of the average** of all the numbers chosen by the participants will be the winner.

For this game, you will compete against the **total number of people** who enter this experiment. Remember that we have sent the invitation to **240** people and we hope that around **160** will respond to the activity.

The winner of game #3 will win $150,000. Your answer:

Game #4 – Choose the average number of others in small groups

The instructions are similar to the previous game.

You must choose any integer you want between 1 and 100, knowing that whoever chooses the number **closest to 2/3 of the average** of all the numbers chosen by the participants in your group will be the winner.

For this game, you will be assigned to a group of **40 people**, and you will compete against them.

The winner of Game #4 in your group will win $40,000. Your answer:

Questions

Please answer the following questions.

Question #1

If Juan can drink a barrel of water in 6 days, and Maria can drink a barrel of water in 12 days, how long will it take them to drink a barrel of water together? ________

Question #2

Miguel placed in both the highest 15th position and the lowest 15th position in his class. How many students are in Miguel's class? ________

Question #3

Oscar buys a pig for $60, sells it for $70, buys it back for $80, and finally sells it for $90. How much money did he earn? ________

Question #4

Simón decides to invest 8,000 USD in the stock market in January 2008. Six months later, on July 17, the stock prices fell by 50%. Fortunately for Simón, between July 17 and October 17, the share price rose 75%. At this point:

☐ Simon has the same money he started with
☐ Simon earned money
☐ Simon lost money

Statements

Please indicate how much you agree with the following statements:

- Assessments in virtual classes do not reveal which students learn more than others

  ☐ Completely agree ☐ Agree ☐ Disagree ☐ Completely disagree

- I'd rather dedicate 9 hours to a home exam and get a good grade, than 3 hours in a face-to-face exam and not knowing if I will perform well or not

  ☐ Completely agree ☐ Agree ☐ Disagree ☐ Completely disagree

- I think it is okay that teachers design several versions of the exam when it is virtual
  ☐ Completely agree ☐ Agree ☐ Disagree ☐ Completely disagree
- I feel that virtual learning has closed the learning gaps between students
  ☐ Completely agree ☐ Agree ☐ Disagree ☐ Completely disagree

Questions about you

Before finishing, we want to ask a few questions about you.

- How old are you? ____________
- What is your GPA? ____________
- What degree(s) are you studying/did you study?
  ☐ Finance and International Trade ☐ Economics ☐ Both
- What semester are you currently in? ____________
- Please leave us your email so we can contact you if you are one of the winners ____________

SM.5 Original protocol (in Spanish) of the incentivized experiment

Pensamiento Estratégico y Evaluación en la Virtualidad

¿Crees poder elegir el menor número entero sin repetición? Participa en este estudio sobre juegos estratégicos, rendimiento académico, y actitudes hacia la evaluación virtual.

Consentimiento Informado

Aquí podrá participar de nuevo en el juego LUPI (Lowest Unique Positive Integer), actividad que realizó en al menos uno de sus exámenes de Econometría Básica. También participará en otro juego similar.

La prueba entera tomará cerca de 15 minutos o menos. Usted participará en los dos juegos, en grupos grandes y grupos pequeños, para un total de cuatro juegos. Haciendo parte de esta prueba podrá participar por cuatro diferentes premios, uno por cada versión del juego. Los juegos de grupos grandes pagan $150.000 al ganador, y los juegos de grupos pequeños pagan $40.000 al ganador de cada grupo.

Su participación es completamente voluntaria, y puede abandonar el test en cualquier momento, sin ninguna penalización o perjuicio. En adición a la información recolectada en la presente prueba, solicitaremos el uso de sus resultados en el examen de Econometría Básica donde jugó LUPI, en específico su respuesta al juego, y sus notas del examen.

Confidencialidad: La información recolectada en esta encuesta podrá ser publicada en un reporte o artículo científico. Sus datos personales no serán compartidos con terceros, y serán utilizados exclusivamente con el fin de relacionar sus respuestas previas con las actuales y poder contactarle en caso de ser uno de los ganadores. Posteriormente está información será totalmente anonimizada y podrá ser almacenada por los investigadores para ser utilizada en estudios futuros.

Esta prueba es parte de un proyecto de investigación sobre las actitudes hacia la evaluación virtual y el rendimiento en evaluaciones y otras pruebas que requieren esfuerzo cognitivo. El responsable científico es César Mantilla de la Universidad del Rosario. En caso de tener preguntas sobre este test por favor envíe un correo a cesar.mantilla@urosario.edu.co.

Sorteo: Se recibirán respuestas a los juegos hasta el 28 de octubre a las 7:00 pm. Posteriormente, se formarán al azar grupos pequeños. Con esta información se calcularán los ganadores, y se publicarán los ganadores junto al código de aleatorización.

Por favor indique debajo que usted leyó la información mostrada arriba, y que usted desea participar en esta prueba en línea.

Leí la información mostrada arriba, y deseo participar en esta prueba en línea. Estoy de acuerdo con el siguiente tratamiento de mi información:

☐ Apruebo el uso de información mía que es recolectada en esta prueba y previamente en clase (respuesta en el juego y nota en el examen).

☐ Apruebo el uso de información mía que es recolectada en esta prueba, pero no de mi información previa en clase (respuesta en el juego y nota en el examen) .

Juego #1 – Escoger el menor entero positivo no repetido en grupos grandes

Usted debe escoger el número entero que desee entre 1 y 100, sabiendo que quién escoja el **menor número positivo no repetido** será el ganador.

Para escoger el número usted enfrenta un *trade-off* (disyuntiva): entre más alto sea el número, menor es el chance de que esté repetido (más chances de ganar), pero también entre más alto sea el número, mayores chances que alguien más diga un número más bajo (menos chances de ganar).

En este juego competirá junto al total de personas que hagan parte de este experimento. Hemos enviado la invitación a **240** personas y esperamos que cerca de **160** respondan la actividad.

El ganador del Juego #1 tendrá una ganancia de $150.000. Su respuesta:

Juego #2 – Escoger el menor entero positivo no repetido en grupos pequeños

Las instrucciones son similares al juego anterior.

Usted debe escoger el número entero que desee entre 1 y 100, sabiendo que quién escoja **el menor número positivo no repetido** será el ganador.

Para escoger el número usted enfrenta un *trade-off* (disyuntiva): entre más alto sea el número, menor es el chance de que esté repetido (más chances de ganar), pero también entre más alto sea el número, mayores chances que alguien más diga un número más bajo (menos chances de ganar).

En este juego, usted será asignado a un grupo de **40 personas**, y competirá junto a ellas.

El ganador del Juego #2 en su grupo obtendrá una ganancia de $40.000. Su respuesta:

Juego #3 – Escoger el número promedio de los demás en grupos grandes

Usted debe escoger el número entero que desee entre 1 y 100, sabiendo que quién escoja el número **más cercano a 2/3 del promedio** de todos los números elegidos por los participantes será el ganador.

En este juego competirá junto al **total de personas** que hagan parte de este experimento. Recuerde que hemos enviado la invitación a **240** personas y esperamos que cerca de **160** respondan la actividad.

El ganador del juego #3 tendrá una ganancia de $150.000. Su respuesta:

Juego #4 - Escoger el número promedio de los demás en grupos pequeños

Las instrucciones son similares al juego anterior.

Usted debe escoger el número entero que desee entre 1 y 100, sabiendo que quién escoja el número **más cercano a 2/3 del promedio** de todos los números elegidos por los participantes de su grupo será el ganador.

En este juego, usted será asignado a un grupo de **40 personas** y competirá junto a ellas.

El ganador del Juego #4 en su grupo tendrá una ganancia de $40.000. Su respuesta:

Preguntas

A continuación le pedimos que responda las siguientes preguntas.

Pregunta #1

Si Juan puede beber un barril de agua en 6 días, y María puede beber un barril de agua en 12 días, ¿cuánto tiempo les va a tomar beber un barril de agua juntos? ______________

Pregunta #2

Miguel quedó tanto en la posición #15 más alta, como en la posición #15 más baja de su clase. ¿Cuántos estudiantes hay en la clase de Miguel? ______________

Pregunta #3

Óscar compra un cerdo a $60, lo vende a $70, lo vuelve a comprar a $80 y finalmente, lo vende a $90. ¿Cuánto dinero ganó? ______________

Pregunta #4

Simón decide invertir 8000 USD en el mercado de acciones en enero de 2008. Seis meses después, el 17 de julio, los precios de las acciones cayeron en un 50%. Por fortuna para Simón, entre el 17 de julio y el 17 de octubre, el precio de las acciones subieron en un 75%. Para este punto Simón:

☐ Tiene el mismo dinero con el que inició
☐ Ha ganado dinero
☐ Ha perdido dinero

Afirmaciones

Indique que tan de acuerdo se encuentra con las siguientes afirmaciones:

- Las evaluaciones en clases virtuales no dejan saber qué estudiantes aprenden más que otros

  ☐ Muy de acuerdo ☐ De acuerdo ☐ En desacuerdo ☐ Muy en desacuerdo

- Prefiero dedicarle 9 horas a un examen para la casa y sacar buena nota, que 3 horas en un examen presencial y no saber si me irá bien o no

☐ Muy de acuerdo ☐ De acuerdo ☐ En desacuerdo ☐ Muy en desacuerdo

- Me parece bien que los profesores diseñen varias versiones del examen cuando la evaluación es virtual

  ☐ Muy de acuerdo ☐ De acuerdo ☐ En desacuerdo ☐ Muy en desacuerdo

- Siento que la virtualidad ha cerrado las brechas de aprendizaje entre estudiantes

  ☐ Muy de acuerdo ☐ De acuerdo ☐ En desacuerdo ☐ Muy en desacuerdo

Preguntas sobre usted

Antes de terminar queremos hacerle algunas preguntas sobre usted.

- ¿Cuántos años tiene usted? ________________
- ¿Cuál es su promedio acumulado? ________________
- ¿Qué carrera(s) estudia/estudió?

  ☐ Finanzas y Comercio Internacional ☐ Economía ☐ Ambas

- ¿Qué semestre cursa actualmente? ________________
- Por favor déjenos su correo electrónico para poder contactarle en caso de ser uno de los ganadores ____
